# Larval and Post-Larval Stages of Pacific Oyster (*Crassostrea gigas*) Are Resistant to Elevated CO_2_

**DOI:** 10.1371/journal.pone.0064147

**Published:** 2013-05-28

**Authors:** Ko W. K. Ginger, Chan B. S. Vera, Dineshram R, Choi K. S. Dennis, Li J. Adela, Ziniu Yu, Vengatesen Thiyagarajan

**Affiliations:** 1 The Swire Institute of Marine Science and School of Biological Sciences, The University of Hong Kong, Hong Kong, SAR; 2 South China Sea Institute of Oceanology, Chinese Academy of Sciences, Guangzhou, China; University of Gothenburg, Sweden

## Abstract

The average pH of surface oceans has decreased by 0.1 unit since industrialization and is expected to decrease by another 0.3–0.7 units before the year 2300 due to the absorption of anthropogenic CO_2_. This human-caused pH change is posing serious threats and challenges to the Pacific oyster (*Crassostrea gigas*), especially to their larval stages. Our knowledge of the effect of reduced pH on *C. gigas* larvae presently relies presumptively on four short-term (<4 days) survival and growth studies. Using multiple physiological measurements and life stages, the effects of long-term (40 days) exposure to pH 8.1, 7.7 and 7.4 on larval shell growth, metamorphosis, respiration and filtration rates at the time of metamorphosis, along with the juvenile shell growth and structure of the *C. gigas*, were examined in this study. The mean survival and growth rates were not affected by pH. The metabolic, feeding and metamorphosis rates of pediveliger larvae were similar, between pH 8.1 and 7.7. The pediveligers at pH 7.4 showed reduced weight-specific metabolic and filtration rates, yet were able to sustain a more rapid post-settlement growth rate. However, no evidence suggested that low pH treatments resulted in alterations to the shell ultrastructures (SEM images) or elemental compositions (i.e., Mg/Ca and Sr/Ca ratios). Thus, larval and post-larval forms of the *C. gigas* in the Yellow Sea are probably resistant to elevated CO_2_ and decreased near-future pH scenarios. The pre-adapted ability to resist a wide range of decreased pH may provide *C. gigas* with the necessary tolerance to withstand rapid pH changes over the coming century.

## Introduction

The entry of anthropogenic CO_2_ into coastal and open oceans is altering carbonate buffering systems and equilibriums and decreasing pH [1], [2]. Fluctuating and decreasing pH levels are threatening oyster population and aquaculture globally and could therefore have severe effects on human welfare [3], [4]. Although there is a lack of direct experimental evidence, recent oyster production declines in aquaculture farms on the North American west coast have been associated with larval exposure to environmentally fluctuating and decreased pH [5]. Such a deleterious effect is expected because larval oyster shells are made of the more soluble form of CaCO_3_, aragonite [6], [7], [8], and because larval developmental processes are sensitive to environmental factors [9], [10], including decreased pH [11], [12], [13], [14], [15], [16], [17], [18], [19], [20].

Oyster species with aquaculture values were notably introduced and established permanently outside their home range and ambient water quality conditions including environmental pH [21]. Microevolutionary processes can occur in a few generations within a population to progressively strengthen individual fitness towards pH fluctuation [22], [23] in their non-native environments, enabling the less-sensitive traits to flourish. Such adaptive tolerance and resistance of oysters introduced to a new environment may provide advantages for certain populations from novel stressors such as decreased pH [24]. For example, while the Australian population of Pacific oyster (*Crassostrea gigas*) has shown depressed shell growth and developmental abnormalities at pH 7.8 [17], the Japanese, American and European populations are resistant to decreased pH [11], [13], [16]. Although the Pacific oyster is one of the important aquacultural and favored introduced species around the world, our knowledge of its larval response to decreased pH is limited to short-term (<4 days) shell growth and survival data obtained from single early life stage (D-shaped larva) [13], [16], [17], [25].

As most larval traits are interconnected, the pH effects on various larval characteristics should be considered together when analyzing the undesirable consequences of a stressor [26]. For an accurate prediction of the effects of multiple pH levels, long-term experiments covering the full length of the development period (i.e., the pre- and post-settlement stages) is required for *C. gigas* [5], [13]. Larval metamorphosis in oysters is rapidly executed (within hours) while larvae switch their calcification mode from aragonite to calcite [7]. This critical life stage process is energetically expensive and highly demanding and represents a bottleneck in the form of recruitment success [27], [28]. This key process and larval physiology at the time of metamorphosis have been overlooked in ocean acidification research [15], [29]. To better understand the consequences of decreasing pH on population success and ecological processes, it is therefore essential to understand what will happen to larvae physiologically at the time of settlement and metamorphosis.

In this study, therefore, the larval response of *C. gigas* from a Yellow Sea-population when exposed to decreased pH levels was evaluated from late-veligers to juveniles using a 40 day long exposure and large-scale (40 L) CO_2_ perturbation experiment. The D-shaped larvae were reared to an early juvenile stage at three pH levels, 8.1, 7.7 and 7.4. Comparative larval developmental (shell growth and metamorphic success), physiological (respiration and filtration rates) and calcification (shell composition and ultrastructure) responses to the pH levels were measured to evaluate the impact of CO_2_-driven decreased pH on the Chinese oyster populations.

## Materials and Methods

### Statement of Ethics

No specific permits were required for the described field sampling. No specific permissions were required for oyster sample collection from Tsingdao (North China) in the Yellow Sea. We also confirm that the location is not privately-owned or protected in any way and the field studies did not involve endangered or protected species.

### Study Organism: Pacific Oysters from the Yellow Sea

Adult Pacific oysters (*Crassostrea gigas*) were collected from the coastal area of Tsingdao (China) in the Yellow Sea (36° 04'N, 120° 22'E) during July 2012 (summer time). The Yellow Sea is a marginal sea of the Pacific Ocean and located in the northern part of the East China Sea. The collection site experiences minimal freshwater influence from nearby rivers and thus have a typical coastal oceanic conditions with mean summer water temperature of 23°C, salinity of 30 to 31 ‰ and pH 8.1 to 8.2 [30], [31]. The collected broodstocks were transported in emersed condition to Hong Kong (22° 15'N, 114° 10'E) by air via Guangzhou (China). In Hong Kong, the adults were acclimatized for 2 days at a pH of 8.1, a salinity of 24‰ and a temperature of 24°C. These salinity and temperature values corresponded to recommended seawater conditions for Pacific oyster larval culture [32]. Natural seawater obtained from the South China Sea adjacent to Hong Kong was used for this experiment. Sperm and eggs obtained from multiple parents (10 male, 20 female; size 10–15 cm) were gently removed from matured gonads and suspended in 0.22 µm filtered seawater (FSW; with a salinity of 24‰), a procedure known as “strip-spawning” [14]. The fertilization of about 12 million eggs was facilitated by gently mixing a small volume of sperm (4–7 ml dense sperm; 1 million eggs) to avoid polyspermy [32], [33]. After 2 h, embryos were separated from the excess sperm using 20 µm nylon mesh and placed in 40 L culture tanks with FSW (density ∼20 embryos ml^−1^). Decreased pH generally negatively affect oyster fertilization, embryonic development and produce heterogeneously sized D- larvae with different degree of impairment [12], [16], [34], [35]. To avoid insufficient number of larvae reaching eye-spot stage for subsequent analysis, therefore, we have started our experiment with matured D-larvae without considering the effect of pH on early larval development. The embryos developed into D-shaped larvae within 16 h at 24°C, pH 8.1, 24‰ (i.e. at ambient control condition). The D-shaped larvae with similar sizes (∼25 µm in diameter) were obtained by washing with a 20 µm nylon mesh and were used in the following pH perturbation experiment. This procedure allows the experiment to begin with similarly sized and fully (without any abnormalities) developed D-shaped larvae.

### pH Perturbation: Experimental Design

There were three pH treatments to examine the effect of pH values that is either currently experienced by these animals due to seasonal or daily fluctuations or expected to be experienced in near-future due to elevated anthropogenic CO_2_. In the oyster collection site, the Yellow Sea, in annual time scale, the sub-surface seawater pH varied between 8.11 to 7.67 due to seasonal changes in salinity and temperature [36]. For example, in winter the pH ranged between 7.67 and 7.92 but in summer it raised to 8.06. The peak summer average pH 8.11 was however found in late summer months (August). Notably, the carbonate system in the oyster collection site (Yellow Sea) is substantially influenced by globally rising anthropogenic CO_2_ when compared to natural CO_2_ input through respiration and mineralization [36]. The pH 8.1 is representing current global surface average as well as average pH at the time of adult oyster collection for this study. The two decreased pH treatments, pH 7.7 and pH 7.4, represented the extreme carbonate system variables (such as *p*CO_2_ and carbonate ion concentration) experienced by oysters today and the average future projected for the year 2100 or beyond [37] [36]. These two decreased pH levels are in fact environmentally realistic in naturally fluctuating estuarine and coastal environments [38], where the Pacific oyster larvae likely to develop. Each treatment had five replicate culture tanks. In the experimental units, e.g., the larval culture tanks, the pH was decreased by bubbling CO_2_-enriched air sufficient to achieve the desired seawater pH values [39], [40]. The CO_2_ concentration in the tanks was adjusted using dual variable area flow meter/controllers (Cole-Parmer Inc.). The seawater pH was monitored as a proxy of *p*CO_2_ level, and together with temperature both readings were recorded twice daily using a pH meter (SG2, Mettler-Toledo), which was calibrated using NBS/NIST standards (at pH 4, pH 7 and pH 10). Aside from the pH and temperature, the salinity, algal food concentrations and total dissolved oxygen levels were measured periodically with a refractometer, haemocytometer and portable dissolved oxygen meter (ORI-1212503 Orion 3-Star, Thermo Fisher Scientific Inc., U.S.), respectively. In both the larval and juvenile culture phase, seawater from the culture tanks was changed every 3 to 4 days, and seawater samples were collected at each time of water change to determine the total alkalinity (TA) using an Alkalinity Titrator (AC-A2, Apollo SciTech's Inc., U.S.) [41]. This frequency of seawater change was generally recommended in oyster larvae hatcheries in China, this is not only due to practical limitation by the common lack of flow-through facilities, it is also regarded to be optimal duration for minimizing environmental shock and physical damage made to oyster larvae during sieving procedure and transportation to a different water mass, respectively. Such frequency of seawater renewal did not cause undesirable health consequence [42]. Immediately after collection, 50 mL water samples were poisoned using 10 µl of 250 mM mercuric chloride, and the whole TA measurement procedure was validated using the certified seawater reference material, with precision of 0.009–0.072, accuracy of 0.26–0.45 (Batch 103, A.G. Dickson, Scripps Institution of Oceanography). The carbonate system parameters in each experimental unit were obtained using the CO2SYS program with the equilibrium constants K1, K2 and KSO4 [43], [44], according to the measured parameters, i.e. temperature, salinity, pH and total alkalinity.

### Pre- and Post-settlement Culture

The D-shaped larvae were equally divided for the 15 experimental units/tanks (3 pH treatments×5 replicates). Each cylindrical plastic culture tank contained 40 L of 0.45 µm filtered natural seawater with 24‰ salinity. Tanks were randomly placed in water baths maintained at 24°C. There were initially 20 larvae per ml^−1^, larval density was monitored for each tank every time during water change. The total volume for culture was adjusted in some tanks to maintain a consistent larval density when necessary. This relatively large-scale (40 L) larval culture system was appropriate to mimic aquaculture hatchery conditions as closely as possible, to get a sufficient number of larvae for various measurements during this relatively long-term (>45 days) experiment and to minimize the considerable larval mortality often experienced in hatchery conditions [35]. During the first 7 days, the early veliger larvae were fed daily *ad libitum* with the monoculture of live small flagellate *Isochrysis galbana* at the final concentration of 5×10^5^ cells ml^−1^ [45]. After 7 days (including the late larval and post-settlement growth periods), animals were cultured using similar methods, but were fed with a mixture of live *I. galbana* and *Chaetoceros gracilis* (late veliger, 5×10^5^ cells ml^−1^ algal mixture *I. galbana* and *C. gracilis* in 8∶2 ratio, twice daily; pediveliger, 8×10^5^ cells ml^−1^, 1∶1 ratio, twice daily; juvenile 10×10^5^ cells ml^−1^, 1∶1 ratio, once daily). Incorporating the diet with the bigger diatoms, *C. gracilis* provides higher lipid content and maximum larval growth rates [46]. Metamorphosis was induced by plastic plates with 7 day old natural multi-species microbial biofilm, metamorphosed juveniles on the plates were kept in 1 L tanks for post-metamorphic growth with similar seawater chemistry, algal proportion and concentrations. From these larval and juvenile cultures, samples were periodically taken for the following measurements.

### Larval Development and Shell Growth

The effects of pH on larval survivorship, the relative proportion of different developmental stages, mean shell growth and frequency distribution of the different size classes were determined on days 11 and 16 post-fertilization. During the seawater change on days 11 and 16, all of the larvae were filtered out using a 50 µm mesh and concentrated into 150–200 ml pH adjusted seawater. To compare survivorship, the concentrated larvae were mixed thoroughly and a subsample (1 ml) was taken from each replicate culture using a wide-mouth micropipette. Samples were immediately fixed in 10% buffered formalin. Under a compound microscope equipped with a digital camera (Leica DFC 280, Leica, Germany), larvae were counted to calculate the percentage of survival based on the number of empty shells (dead at the time of fixation) and shells with larval tissue inside (alive at the time of fixation). Since all the experimental pH level had aragonite saturation stage, the shells of dead animals shall be well retained in the culture tank. At the same time, the larvae were classified according to different developmental stages and their numbers were recorded. The developmental stages were identified using the shell morphology and the presence of D-shaped, early umbo, late umbo and pediveliger stages with eyespots [47]. All of the shells were then photographed at a 63×magnification. Using the picture, projected shell areas of the right valves of randomly chosen larvae were measured for growth analysis using a Leica QWin V3 data processing tool [48]. The shell growth rates of the early juveniles were measured using the following procedure. The locations of randomly chosen post-settlement individuals on the substrates deployed in each treatment tank were marked with permanent ink to enable repeated records of the same individual. The area of each juvenile’s right valve (outer shell) was measured over time using Leica QWin V3, which was subsequently used to calculate its growth rate. Juvenile growth rates were measured on days 5 and 12 post-settlement. For each sample, there were 25 to 100 larvae, or one to 18 individuals for the early juveniles. The cumulative percentage abundance were plotted against shell surface area categories in each replicates, the curves were fitted with three parameter Gompertz asymmetric nonlinear growth model using non-linear curve fitting option in the statistical software Sigmaplot (version 12.0) [49]. The shell area at the greatest slope of the curve, i.e. the inflection point, x and y (Eqn. 2), were calculated based on the statistical parameters (a, b, k) obtained from the least squares program for the nonlinear Gompertz equation and model (Eqn. 1). The effect of pH on these quantitative parameters in each of the sampling dates for larvae (day 11 and 16) and juvenile (day 5 and day 12) shell area values were then compared using one way ANOVA. In rare occasion observed in one of the replicates in pH 7.7 and pH 7.4 treatments for day 11 larvae did not converge to the Gompertz model, and were excluded from the analysis. Even then, there were four replicates or above in all sampling dates for analyses of pH effects.
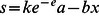
(1)

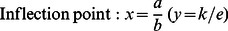
(2)


### Larval Scope for Growth and Metamorphosis

As soon as some larvae developed into the pediveliger stage with an eyespot and a foot (about 10%) and were competent to attach and metamorphose into early juveniles, they were sieved out using a 280 µm mesh. The required numbers of pediveligers (about 600 per tank) were obtained from each tank irrespective of pH treatments and culture tanks. The similar size (about 280 µm long), developmental stage (pediveliger stage) and age (16 days post-fertilization) of the larval cohort permitted us to examine the effects of pH on the larval metamorphosis, respiration and filtration rates without interference from potential confounding factors such as the virtual age effect or delayed (or enhanced) growth due to pH treatments [50], [51]. Nevertheless, this procedure has a drawback, i.e. the pediveliger larvae used for the following measurements did not represent the entire larval population and they may have come from the fast-growing larval population within a given treatment.

A simplified representation of the larval energy expenditure and intake, in terms of respiration and feeding, were used to estimate how scope for growth may be influenced by pH. Respiration (oxygen consumption) rates were continuously measured using polarographic oxygen sensor (POS) respirometer [52]. The six-channel POS dissolved-oxygen measuring system (Strathkelvin Instruments, Glasgow, U.K.) with Strathkelvin 1302 microcathode oxygen electrodes were connected with a standard Strathkelvin RC650 respirometer [53]. The electrodes were calibrated against oxygen-free seawater (i.e., solution with 4% sodium sulphite) and air-saturated FSW at each pH treatment. Exactly 200 larvae were added into a respiration chamber (volume of 3 ml) for each replicate (n = 3). Such a larval density had minimal effects on the larvae’s health and allowed sensitive detection of the respiration rate [52]. Each respiration chamber had a calibrated polarographic oxygen sensor (Model 1302, Strathkelvin), and an adjustable air tight cap, 2 ml of FSW and a magnetic stirrer with 600 rpm, air bubbles were expelled out of the respiration chamber before the start of the measurement. The pH in each chamber was pre-adjusted in correspondence with its larval treatment condition. The larvae were acclimated for 1 h in respiration chambers before measurement. The chamber temperature was maintained at the larval culture temperature through a circulating water bath (24°C). The oxygen consumption levels were measured for 1 h. A control chamber corresponding pH water without larvae were used to monitor bacteria O_2_ consumption, such values of O_2_ consumption (about 6%) were used as the background values for the corresponding pH measurement. Additionally, to minimize this noise created from bacterial respiration, seawater, respiration chamber and all apparatus were sterilized in advance. After measurement, the larvae were removed from the chambers, rinsed with distilled water to remove salt and seawater and grouped in pre-weighed aluminum foil sachets to measure the ash-free dry weight (AFDW). The foils were first dried in an oven at 50°C for 48 h and were then weighed to the nearest 0.001 mg (dry weight) using a microbalance (R200D, Sartorius, U.S.). The foil sachets were then placed in a muffle furnace at 550°C for 16 h and reweighed (ash weight). The AFDW was calculated as the dry weight (inorganic plus organic contents) minus the ash weight (inorganic contents only). The mass specific oxygen consumption rates of the larvae were expressed as the microliters of oxygen consumed per milligram of AFDW per hour [52]. Only three randomly selected replicates per pH treatment were used due to the logistic challenges.

The effect of pH on the larval filtration (feeding) rate was determined in a 50 ml centrifuge tube that contained 200 larvae, 30 ml of FSW and 4×10^6^ cells ml^−1^ of *I. galbana* [54]. The pH and temperature were pre-adjusted corresponding to their larval treatment. The larvae were incubated in the centrifuge tube for 15 h, every 1–2 h, centrifuge tube were inverted to facilitate algal mixing. From each centrifuge tube, 1 ml of seawater samples was fixed (algal cell division and consumption quenched with 400 µl 10% buffered formalin) for the algal concentration measurement using a Coulter counter (Coulter Counter® Multisizer II) before and after the experiment, together with a blank chamber in the absence of larvae [55]. After the experiment, the larval AFDW was determined as described in the previous paragraph. The weight-specific filtration rate was calculated from the exponential decrease in *I. galbana* concentration during the 15 h feeding period ({[Vx(ln C_0_-ln C_1_)]/t}/AFDW; V = 30 ml; t = 15 h; C_0_ = initial algae concentration; C_1_ = final algae concentration) and expressed as the milliliters of water filtered per hour (ml mg^−1^h^−1^). There were five replicates per pH treatment. Autoclaved seawater was used throughout the experiment.

From larval respiration and filtration rates, the energy available for growth and development, i.e. the scope for growth (SfG), was calculated by estimating the larval energy input and output. Respiration rates were converted to energy equivalent using the standard conversion value of 0.45 J mgh^−1^ [56]. The larval clearance rate (feeding) was converted to energy equivalent of 23 J mgh^−1^ per mg of particulate organic matter [57] by using the larval food *Isochrysis galbana* with an estimated cell mass of 20.8 pg cell^−1^ [58], average cell size of 45 µm^3^ cell^−1^, a carbon content of 9.37 pg C cell^−1^, and with an estimated dry weight of 0.51 gL^−1^. Scope for growth was then calculated from energy intake (I) and respiratory energy loss (R) as SfG = I−R. As excretion rates of oyster larvae were not included in the calculation, our SfG measurements may have been overestimated across treatments. The percentage metamorphosis of pediveligers was determined using the following bioassay. Under a dissection microscope, a known amount (∼200) of larvae was transferred into a 1 L plastic bioassay vessel that contained 750 ml of FSW and a plastic sheet covered with biofilm (i.e., 5-day-old natural microbial film developed in an outdoor seawater aquarium). Oyster larvae are known to be stimulated by chemical cues from biofilm-developed surfaces, and are induced to settle and metamorphose [59]. The pH and temperature levels for these bioassay vessels were pre-adjusted corresponding to their larval treatment conditions. The percentage of larvae that attached and metamorphosed on each biofilm surface was counted after 24 h. The larvae seldom attached on the clean wall of the bioassay container according to observation. Measurements from all five replicate tanks were taken in each pH treatment.

### Juvenile Shell Area Ratio, Thickness, Ultrastructure and Composition

After 24 days of post-settlement growth, all juveniles were carefully detached from the substrate and preserved in 75% ethanol to measure their shell area ratios, thicknesses, cross-sectional ultrastructures and compositions. Whole juvenile shell valves (∼5 individuals from each replicate tank) were mounted onto aluminum stubs with carbon tape and were examined under a scanning electron microscope (SEM) (Hitachi S-3400N VP SEM), which allowed clear observation of the prodissoconch II-dissoconch (PII-D) boundary, i.e., the boundary marking the distinctive prodissoconch and dissoconch regions as in larval and juvenile calcification, respectively [60]. The area ratios between the whole juvenile shell (including both the prodissoconch and dissoconch regions) and larval prodissoconch area were quantified to indicate the net shell growth after metamorphosis using the Image J program (Image J 1.45s, NIH, U.S.).

The shell thicknesses and ultrastructures were compared as visualized from the cross-sectional specimen. The juvenile shells were embedded in resin using silicon molds suitable for ultramicrotomy. The excess resin was carefully trimmed away from the embedded specimens, and a cut passing through the shell hinge was created for each section [48], [61]. The cross-sectional surface was subsequently smoothened by ultramicrotome sectioning, and a glass and diamond knife were then used consecutively to remove thin (down to ∼70 nm thick) sections (Ultracut S, Leica, Germany). The shell sections were treated with 2 min of etching using a 0.5 M EDTA solution to reveal the topographical details of the ultrastructures. The etched surfaces were rinsed with double distilled water and air dried, and the specimens were then mounted onto an aluminum stub with carbon tape and surrounded with silver paint to prevent electron charging. Finally, the specimens were sputter coated with a gold-palladium alloy (∼50 nm) prior to imaging using a Leo 1530 FEG SEM.

The elemental ratios for magnesium/calcium (Mg/Ca) and strontium/calcium (Sr/Ca) in the juvenile shells were determined to evaluate the effects of pH on their shell compositions. The relative intensities were simultaneously measured for calcium (Ca, 396.847 nm), magnesium (Mg, 285.213 nm) and strontium (Sr, 407.771 nm) using inductively coupled plasma-optical emission spectrophotometry (ICP-OES, PE Optima 8300). The harvested shell valves (one to five juveniles from each replicate) were immersed with 5% bleach (NaOCl, CloroxTM) for about 30 min to remove soft tissues. The cleaned shell valves were rinsed twice with double-distilled water prior to acid digestion with 2% nitric acid. All of the containers used for sample handling were acid washed in 10% v/v HCl overnight, rinsed twice with double-distilled water and dried at 80°C before use. Samples in different dilutions (1-, 10- and 100-fold dilutions) were prepared to ensure that each metal was quantified within the concentration range of the standard calibration curve [62]. Shell measurements from all five replicate tanks were taken in each pH treatment.

### Statistical Analysis

Before analysis, data were checked for the normality and the homogeneity of variance using the Shapiro-Wilk test and Levene’s test, respectively. The percentage metamorphosis data were arcsine transformed, and all of the other measurements were square root transformed to achieve variance homogeneity. After the transformations, all of the datasets passed the aforementioned two assumptions and were subsequently examined using one-way ANOVA. In cases where a significant pH effect was detected, Tukey’s multiple comparison tests were used to differentiate between the three pH groups. Power analyses were performed when no statistical significance was observed. The sample size for all experimental groups was three to five, with each sample representing the average measurement of one to 200 oyster larvae/juveniles in a single replicate tank. The data were presented as mean±SD. The differences were considered significant if the probability of a Type I error was less than 0.05.

## Results

### CO_2_ Perturbation and Carbonate System

**Figure 1:**
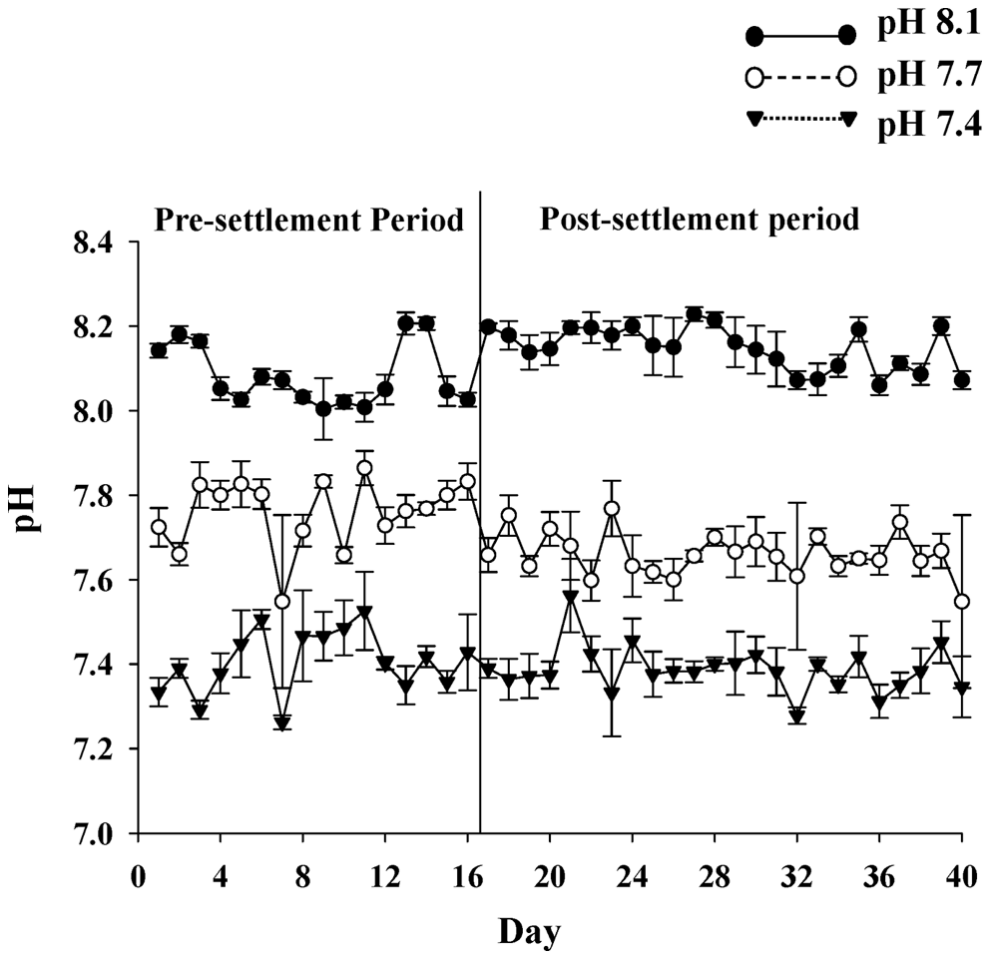
Mean (± S.D of 5 replicate cultures) pH (NBS scale) change during the pre-settlement and post-settlement (juvenile) oyster growth experiment.

The carbonate system for each pH treatment is described in Table 1. The control (pH 8.02±0.02, mean ± SD) and two levels of low pH treatment (pH 7.66±0.02; pH 7.49±0.06) were maintained at significantly different pH and aragonite saturation states (Ω_A_) (ANOVA: pH, F_2,12_ = 229.786, p<0.001; Ω_A_, F_2,12_ = 167.711, p<0.001). There were no significant differences in salinity, temperature or total alkalinity (ANOVA: salinity, F_2,12_ = 0.0167, p>0.05; temperature, F_2,12_ = 0.300, p>0.05; total alkalinity, F_2,12_ = 0.710, p>0.05; Tukey’s post hoc test; p>0.05 for all experimental group comparisons). The daily pH measurements indicated that the carbonate systems within and among the treatment tanks were very stable throughout the course of this experiment (Figure 1).

**Table 1:** Representative values of carbonate system parameters in the three pH treatment culture containers during larval (pre-settlement) and juvenile (post-settlement) growth period.

	Measured				Calculated			
pH	pH	Temp	Salinity	TA	*p*CO_2_	CO_3_^2−^		
Treatments	_(NBS scale)_	(°C)	(‰)	(mmol kg^−1^)	(µatm)	(µmol kg^−1^)	Ω_Cal_	Ω_Ag_
Pre-settlement								
pH 8.1	8.02±0.02	24.6±0.3	24.8±0.4	2.04±0.13	622	109	2.85	1.81
pH 7.7	7.66±0.02	24.7±0.1	24.8±0.8	1.97±0.03	1497	50	1.29	0.82
pH 7.4	7.49±0.06	24.7±0.0	24.6±0.5	2.05±0.14	2386	36	0.93	0.59
Post-settlement								
pH 8.1	8.05±0.02	24.4±0.2	24.5±0.1	1.85±0.34	524	104	2.70	1.72
pH 7.7	7.67±0.02	24.4±0.1	24.3±0.3	1.92±0.75	1429	48	1.26	0.80
pH 7.4	7.37±0.02	24.5±0.1	24.6±0.5	1.91±0.27	2874	25	0.66	0.42

Samples for the pre-settlement carbonate system data were recorded at the time of larval sample collection for the metamorphosis assay, respiration and filtration rate measurements (the eyespot stage with a well-developed foot or pediveliger stage). Samples for the post-settlement carbonate system data were collected at the end of experiment. The partial pressure of carbon dioxide (*p*CO_2_), carbonate ion concentration (CO_3_^2−^), calcite saturation state (Ω_Cal_) and aragonite saturation state (Ω_Ag_) were calculated using CO2SYS.

### Larval Development and Survival

**Figure 2:**
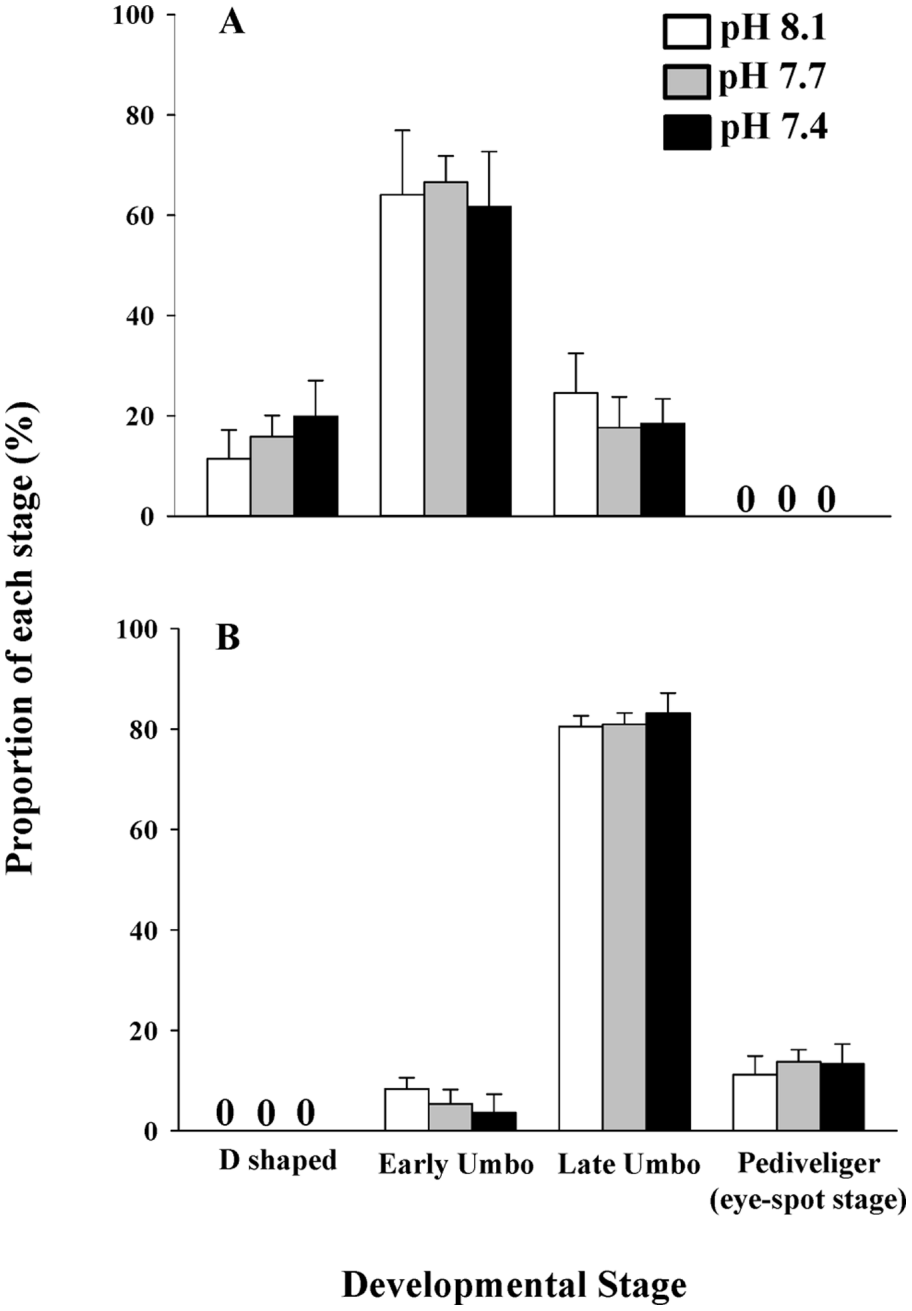
Effects of pH on different developmental stages of the Pacific oyster (*Crassostrea gigas*) on day 11 (A) and day 16 (B) post-fertilization. Each data point represents the mean ± SD of five replicate cultures. For each replicate measurement, 20 to 200 larval samples were used.

The abundance of each development stage was similar among the three pH treatments on both days 11 and 16, i.e., the D-shaped (day 11: F_2,12_ = 2.647, p>0.05, power = 0.11), early umbo (day 11: F_2,12_ = 0.284, p>0.05, power = 0.75; day 16: F_2,12_ = 3.222, p>0.05, power = 0.76), late umbo (day 11: F_2,12_ = 1.709, p>0.05, power = 0.22; day 16: F_2,12_ = 1.179, p>0.05, power = 0.34) and pediveliger stages (day 16: F_2,12_ = 0.786, p>0.05, power = 0.48) (Figures 2A and 2B). After 16 days of pre-settlement growth, <20% of the larvae had reached the settlement stage (i.e., pediveliger). Mean larval survivorship measurements, as shown from the live/dead larvae ratio, showed no observable pH effects for day 0 to 11 or day 11 to 16 (day 11: F_2,12_ = 2.919, p>0.05; day 16: F_2,12_ = 0.561, p>0.05). The survival percentages (mean ± SD) of the oyster larvae in pH 8.1, 7.7 and 7.4 were 75.71±15.26%, 82.68±3.59% and 81.17±2.45% (day 11); and were 87.13±6.19%, 88.77±3.16% and 77.95±8.96% (day 16), respectively.

### Larval Shell Growth

**Figure 3:**
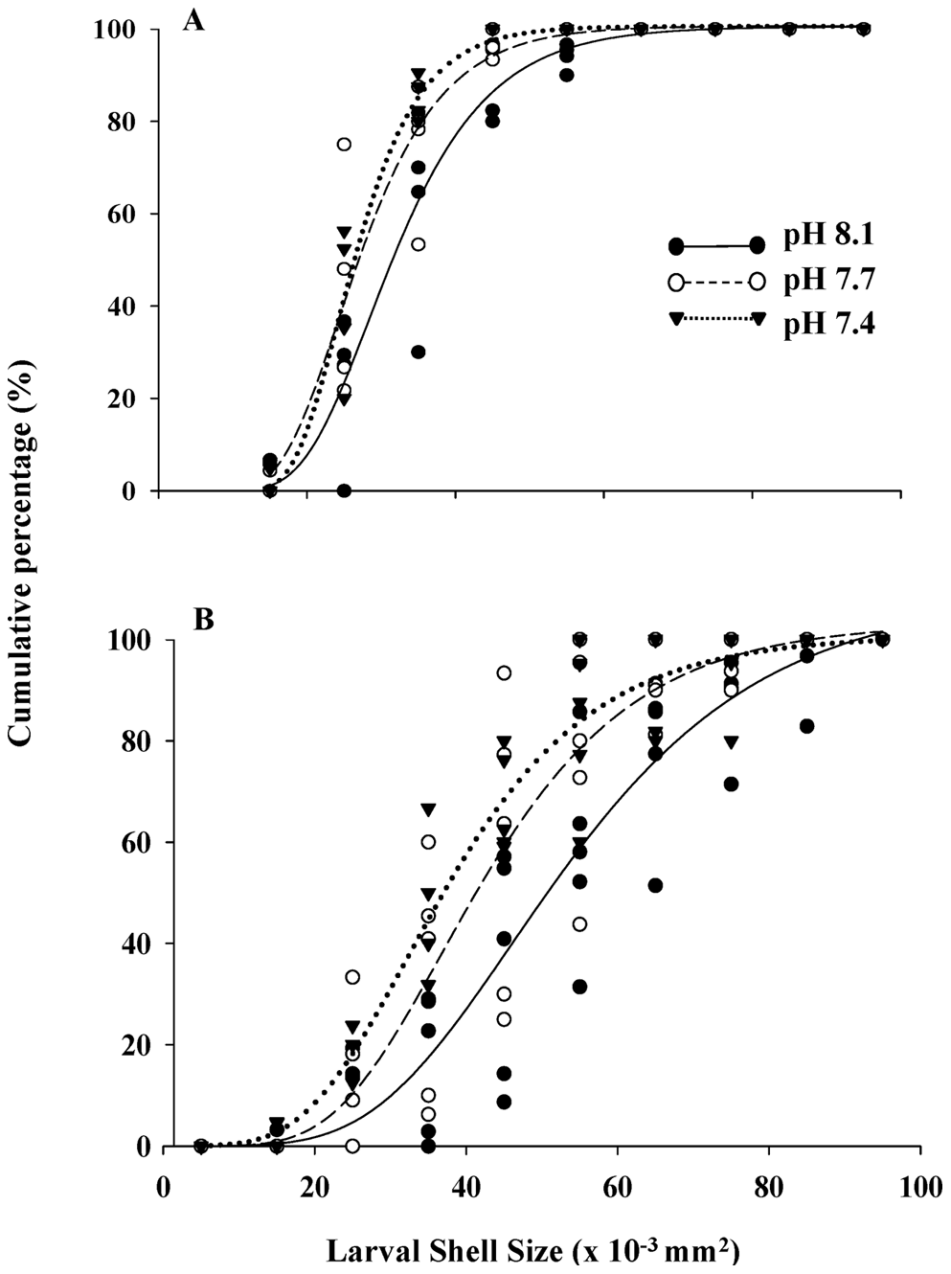
Cumulative percent-frequency distribution of the Pacific oyster (*Crassostrea gigas*) larval shell size at three pH levels (pH 8.1, 7.7 and 7.4) on day 11 (A) and day 16 (B) post-fertilization.

At ambient pH 8.1, the larval shell area increased at a rate of 5.4±1.7×10^−7^ mm^2^ day^−1^ until day 11, but shell growth between days 11 and 16 slowed to 2.6±0.9×10^−7^ mm^2^ day^−1^. As a consequence, each larva gained an average shell area of 2.1–2.5×10^−2^ mm^2^ on day 11 and 4.5–5.7×10^−2^ mm^2^ on day 16. This shell increment over time was not affected by the pH level (day 11: F_2,12_ = 0.998, p>0.05, power = 0.40; day 16: F_2,12_ = 2.894, p>0.05, power = 0.10). The larval size distribution was further examined in a cumulative percentage plot fitted with a non-linear Gompertz model (Figure 3). On day 11, the cumulative percentage curve shared similar inflection points regardless of pH treatments (Figure 3A, inflection point pH 8.1 = 23.58±5.06×10^−3^ mm^2^; pH 7.7 = 19.90±3.85×10^−3^ mm^2^ and pH 7.4 = 19.12±2.71×10^−3^ mm^2^). At Day 16, the shell area at the inflection point was smaller due to the impacts of both decreased pH treatments (Figure 3B, pH 8.1 = 46.90±10.09×10^−3^ mm^2^; pH 7.7 = 38.13±9.86×10^−3^ mm^2^ and pH 7.4 = 33.30±3.63×10^−3^ mm^2^).

### Respiration, Feeding Efficiency and Metamorphosis of Pediveliger

**Figure 4:**
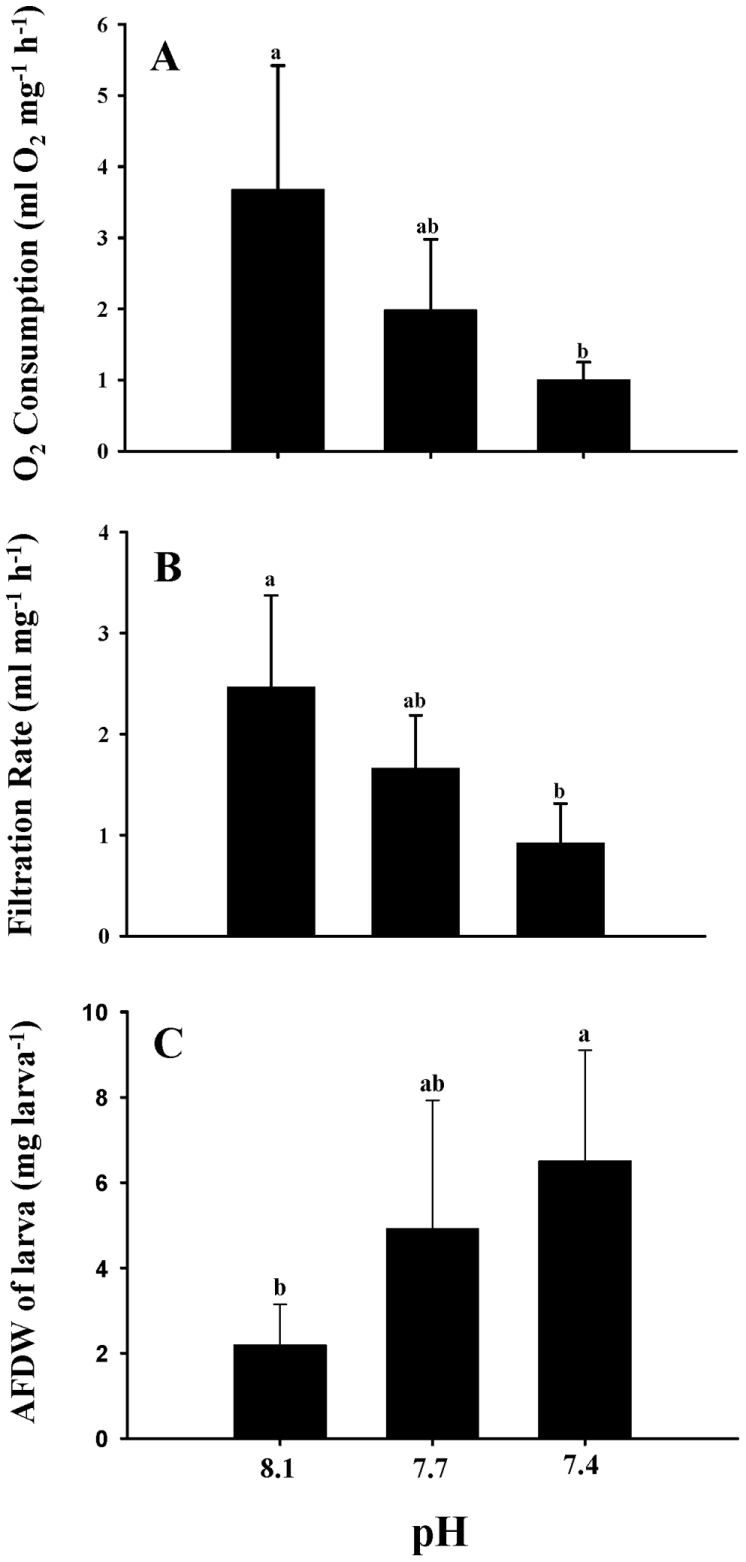
Respiration or oxygen consumption (A), feeding or filtration (B), and organic tissue weight or ash-free dry weight (C) of the pediveliger larvae of the Pacific oyster (*Crassostrea gigas*) cultured at three pH levels (pH 8.1, 7.7 and 7.4). Pediveliger larvae with similar shell size (∼280 µm in length) and age (16 day post-fertilization) from all three pH levels were used simultaneously for each measurement. Each data point represents the mean ± SD of five replicate cultures except respiration, where there were only three randomly selected replicates. For each replicate measurement, 200 larval samples were used. Statistically different mean values (Tukey’s multiple comparison test results) are indicated with different letters.

The respiration (oxygen consumption) and feeding (food particle filtration) rates showed a tendency to decrease with the decreasing pH (Figures 4A and 4B, respectively). While the weight-specific respiration and filtration rates at pH 7.7 were statistically similar to the ambient pH 8.1, they were significantly reduced at the extreme treatment pH 7.4 (respiration: F_2,8_ = 4.485, p<0.05; feeding: F_2,8_ = 4.526, p<0.05). The organic tissue weight (AFDW) or organic content of the larvae raised at pH 7.4 were significantly higher than those raised at pH 8.1 or 7.7 (F_2,8_ = 4.331, p<0.05) (Figure 4C). Once the larvae developed into the pediveliger stage on day 16, 20–30% of them successfully attached and metamorphosed into the juvenile stage within 24 h irrespective of treatment (F_2,12_ = 1.371, p>0.05, power = 0.26). The pH did not significantly affect the scope for growth (SfG) (F_2,8_ = 1.57, p>0.05, power = 0.27). Nevertheless, the SfG calculation and measurements showed high variability among replicates within a treatment, which prevent us to make firm conclusion.

### Post-settlement Growth

**Figure 5:**
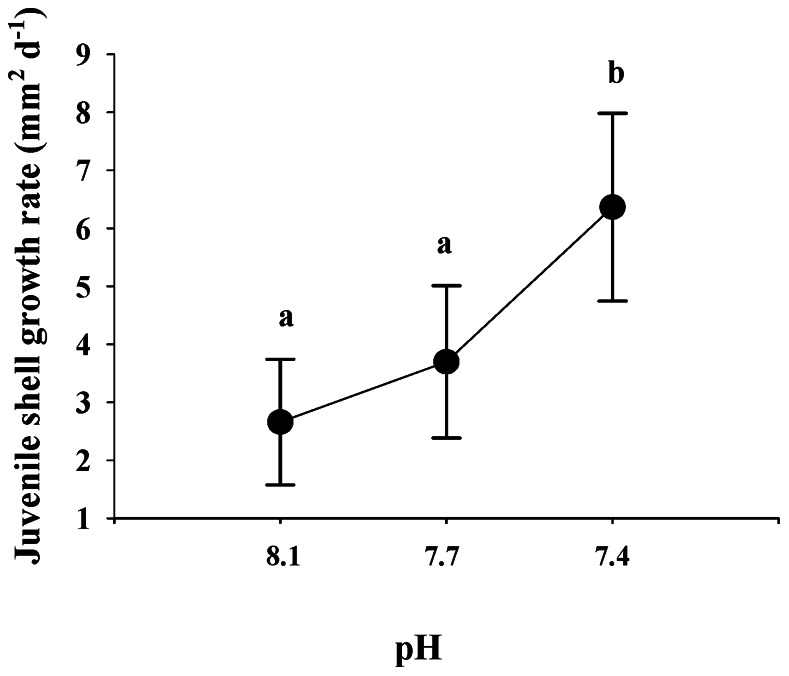
Effects of pH on the post-settlement (early juvenile) shell growth rate of the Pacific oyster (*Crassostrea gigas*). The growth rates for each pH were calculated from days 5 to 12 post-settlement. Each data point represents the mean ± SD of three replicate cultures. For each replicate measurement, one to 20 juvenile samples were used. The statistically different mean values (Tukey’s multiple comparison test results) are indicated with different letters.

**Figure 6:**
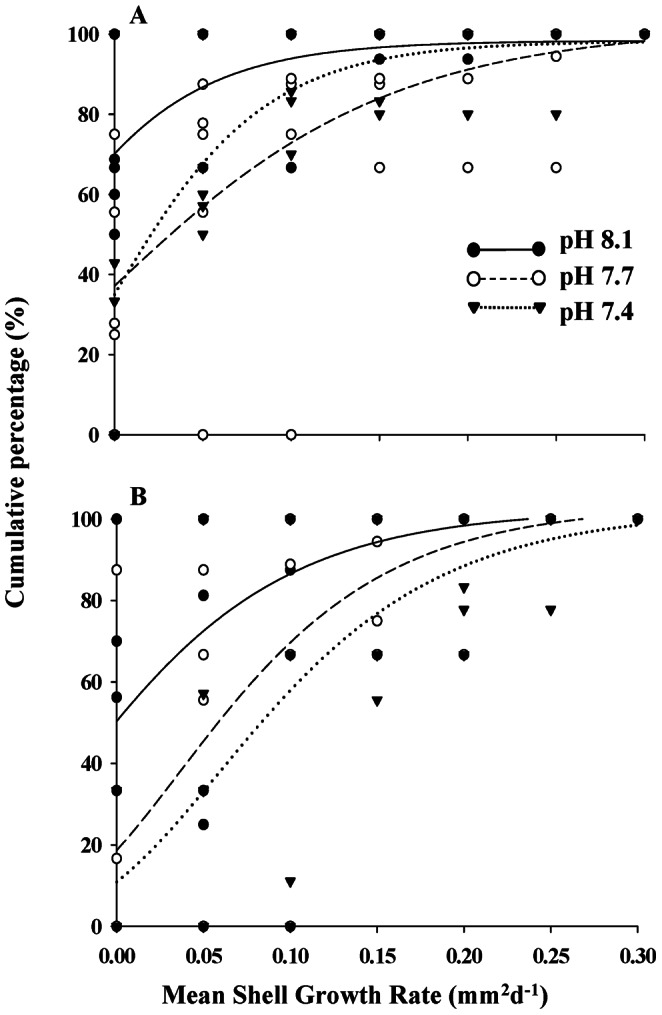
Cumulative percent-frequency distribution of the Pacific oyster (*Crassostrea gigas*) juvenile shell growth rates at three pH levels (pH 8.1, 7.7 and 7.4) on day 5 (A) and day 12 (B) post-settlement.

**Figure 7:**
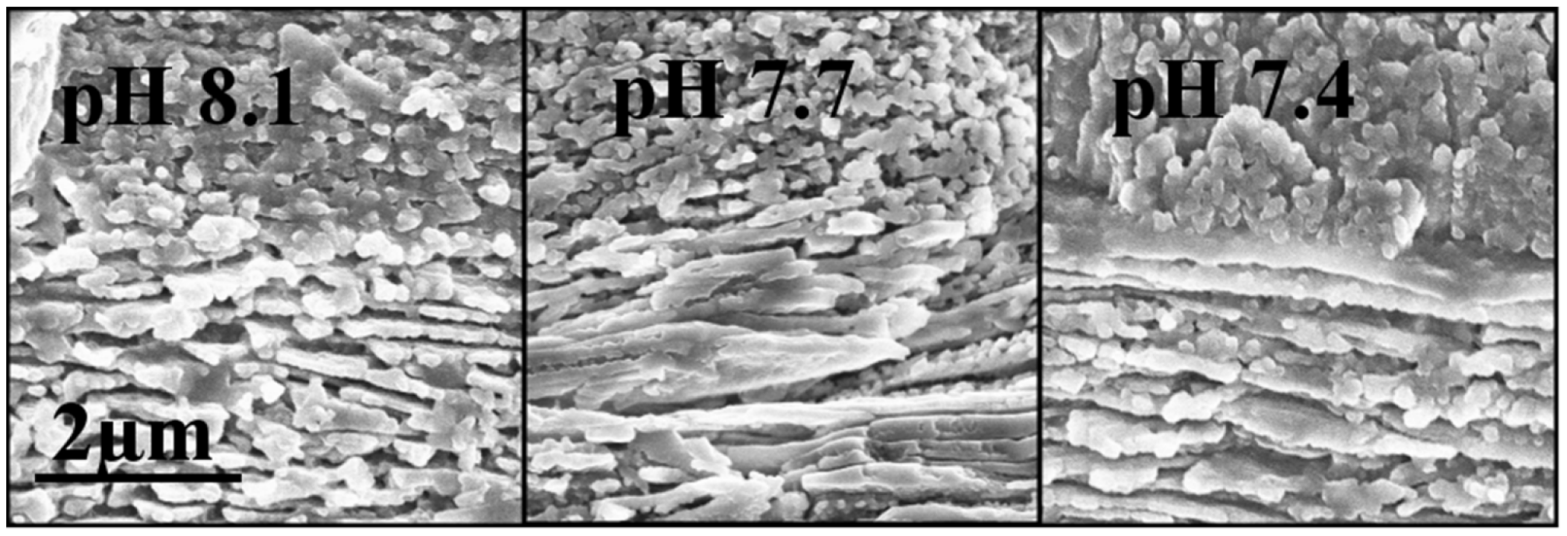
SEM images showing the ultrastructure of the early juvenile shells (42 day post-settlement) of the Pacific oyster (*Crassostrea gigas*) cultured at three pH levels (pH 8.1, 7.7 and 7.4).

The mean shell growth rate, calculated from measurements taken at 5 and 12 days post-settlement, was ∼2.6 mm^2^ day^−1^ at the ambient pH 8.1. While the juvenile growth rate in pH 7.7 was statistically similar to pH 8.1, the juveniles in pH 7.4 grew significantly faster (∼6.3 mm^2^ day^−1^) than their counterparts in pH 8.1 (F_2,11_ = 1.557, p<0.05; Figure 5). Such a rapid shell growth rate occurred only during the late juvenile growth period from days 5 to 12 post-settlement. The juvenile size composition was further examined in a cumulative percentage plot fitted with a non-linear Gompertz model (Figures 6A and 6B). At Day 5, the cumulative percentage curve shared similar inflection points regardless of pH treatments (Figure 7A, inflection point pH 8.1 were all less valid values; pH 7.7 = 0.016±0.083 mm^2^; pH 7.4 = 0.008±0.013 mm^2^). At Day 12, the shell area at the inflection point was smaller due to the impacts of both decreased pH treatments (Figure 6B, pH 8.1 = 0.022 mm^2^; pH 7.7 = 0.020 mm^2^; pH 7.4 = 0.038 mm^2^). The higher ratio between the total shell area (i.e., larval plus juvenile shell) and the larval shell area alone (prodissoconch) further corroborated that the low pH 7.4 induced a more rapid juvenile calcification of the calcitic dissoconch shell. Nevertheless, the pH did not affect the juvenile shell thickness, which had a mean thickness of 16.94±4.74 µm (F_2,10_ = 0.133, p>0.05, power = 0.25).

### Ultrastructure and Elemental Composition of a Juvenile Shell

Two textural layers were commonly observed from the SEM images of the juvenile shells, including the outer layers of the granular structures and the inner layers of the foliate structures. The structure and crystal orientations appeared unaffected by low pH treatments and shared very similar shell microstructures and integrities (Figure 7). Further, the pH did not significantly affect the incorporation of both Mg and Sr into the aragonite larval shells. As a consequence, the Mg/Ca (F_2,12_ = 0.034, p>0.05, power = 0.11) and Sr/Ca ratios (F_2,12_ = 2.554, p>0.05, power = 0.71) in the early juvenile shells were statistically similar across the three pH treatments.

## Discussion

### Pre- and Post-settlement Growth: The Effects of pH

A decrease in pH did not affect either the mean larval shell size or relative composition of the different development stages on days 11 or 16 pre-settlement. The pH 7.4 treatment resulted in a more rapid post-settlement growth, suggesting a selective pressure posed by decreased pH on the more tolerant individuals within a population. Pre- and post-settlement growth and survival seemed to be resistant to the projected decrease in pH. Although the effects of pH on fertilization success and embryonic development were not examined, both responses have been found to be quite unaffected by a decreased pH of 7.6 in the western Sweden population [14]. A similar insignificant pH effect on fertilization and embryo development has been found in the Oregon [5]and Northwestern U.S. populations [25]. Nevertheless, oyster embryo growth as a function of pH deserves further study. With over 45 days of pH exposure involving multiple developmental stages, this study further suggests that the larval growth and development of Yellow-Sea-population may be resistant and tolerant to the detrimental effects of a decreased pH (7.4). Similar to our results, the pH did not affect the larval growth and development of the Suminoe oyster at pH 7.8 [11] or the Portuguese oyster at pH 7.5 [35].

Once hatched from embryos, oyster larvae feed and develop in an estuary and coastal area where water pH naturally varies on a daily basis, sometimes reaching pH values below 7.4 [63], [64]. It appears that oyster larvae from the Yellow Sea population have apparently evolved a physiological tolerance to pH 7.4 through exposure to wide ranges of natural pH variation in their estuarine and coastal habitats. Similarly, sea urchins (*Strongylocentrotus franciscanus*), corals (*Acropora millepora*) and red abalone (*Haliotis rufescens*) have been shown to have adequate genetic diversity at population levels to resist pH decreases [65], [66], [67]. Natural selection, genetic variability, phenotypic plasticity and pre-adaptation at the population level have similarly appeared to protect several echinoids [68], [69] and ectothermic marine invertebrates [70] from pH decreases. Alternatively, during the 16 days of exposure to deceased external pH the larvae may have developed physiological strategies to allocate extra energy for pH homeostasis at decreased pH external environments without compromising calcification and development. Nevertheless, to understand larval energy allocation and to predict changes across life stages in responding to environmental stressors, dynamic energy budget (DEB) models have been developed for several marine species [71] including the *C. gigas* larvae [45]. In the future, these models may provide predictions of where and when individuals within a population will flourish or perish under decreased pH.

In contrast to our results, the survival and growth of early larval forms significantly decreased at pH 7.8 in the Sydney rock oyster [20], [72], Eastern oyster [11], [19], [73] and Olympia oyster [15]. A carbonate ion concentration below 100 µmol kg^−1^ (pH 7.7 and 7.4 in this study; Table 1) caused significantly decreased shell growth, shell calcium incorporation and survival of the D-shaped larvae in the European Pacific oyster [13]. A striking miniaturizing effect on mid-stage larvae by pH reduction (between 7.6 and 8.2) was observed in the U.S.-population Pacific oysters (Oregon) [5]. The calcification and growth rate of developing embryos in the Japanese population similarly decreased significantly at pH 7.4 [16]. Due to technical limitations (see materials and method section), this study did not examine the impact of decreased pH on early life stages, i.e. fertilization and embryos. If there is any such early mortality and delayed growth, our results may have slightly underestimated the impact of decreased pH on overall larval life of the Yellow Sea oyster population. In this study, larvae fed ad libitum but in nature food limitation may exacerbate pH effect. For example, mussels produce weaker shell growth under limited food availability in decreased pH [55]. Nevertheless, the tolerance of oyster larvae to a decreasing pH environment seems to be species- and population-specific. Therefore, the holistic consequence of this commercial species in the ecosystem and aquaculture food production may be more unpredictable than previously thought [18].

### Larval Performance at the Time of Metamorphosis: The Effects of pH

The decrease in the *C. gigas* larval respiration rate along with the decrease in pH observed during this study correspond to results obtained in previous studies of peanut worms, brittle stars, mussels and coral larvae [74], [75], [76], [77], [78]. When the data were normalized to the larval organic weight (AFDW), both respiration and feeding decreased at pH 7.4. Short-term metabolic depression to a decreased pH is a well-known natural occurrence. Metabolic depression can be a direct result of cellular hypercapnic conditions [78], when low intracellular pH conditions inhibit enzyme activity [79]. Energy-demanding ion exchange processes such as the Na^+^/H^+^ exchange and Na^+^-dependent Cl^−/^HCO_3_^−^ exchange contribute to a significant increase in metabolic costs in responding to extracellular pH reduction [51]. Despite this increased energy requirement, proton-producing metabolic processes are suppressed in range of marine invertebrates in an attempt to slow down cellular acidosis [80], [81]. However, such a response is also associated with deleterious consequences, including amino acid catabolism, modified amino acid metabolism and reduced protein synthesis rates [82]. For example, muscle degradation of the brittle star was found to occur at a decreased pH (7.7 to 6.8) despite its increased respiration rate [83]. A similar positive response was also seen in juvenile oysters [73], copepods [84], sea stars [85] and feeding sea urchin larvae [86].

Similar to respiration, the weight-specific filtration rate of the *C. gigas* larvae in the present study was significantly suppressed at pH 7.4. Notably, decreased respiration and feeding at pH 7.4 were evident only when data normalized to larval organic weight (AFDW) but not to body shell size or individuals. Treatment specific variation in larval shell organic weight, which is not metabolically active, may have accounted for the decreased AFDW at pH 7.4. Therefore, we may have slightly underestimated the respiration and filtration rates at pH 7.4. It should be noted that the AFDW shown here is representing only larvae used for respiration but not the larval population in culture. Nevertheless, the decreasing respiration and feeding rates with decreasing pH was evident independent on correction by individuals or AFDW (Figures 4A and B). However, further studies are required to confirm this.

Despite the suppressed filtration rate and metabolic rate, we found no effects on metamorphosis at a reduced pH of 7.7 or 7.4. Similar to our results, there was no effect on the metamorphosis response of the hard clam (*Mercenaria mercenaria*) when subjected to pH 7.6 [87]. Several recent coral studies have demonstrated no effect of a decreased pH on metamorphosis [29], [88]. The competent larvae cultured at pH 7.4 in the present study had a notably higher amount of organic tissue (AFDW in Figure 4C: a proxy of the larval physiological condition). As a larva’s metamorphosis efficiency generally increases along with its organic content or physiological quality [89], we therefore expected a higher percentage of metamorphosis at pH 7.4 due to the higher AFDW. Because oyster larvae are frequently facing natural starvation, they have considerable plasticity in adapting to a stressful environment with a depressed metabolic rate [90]. Nevertheless, the available extra energy or metabolically active organic tissue (AFDW) at pH 7.4 may have helped them to speed up the post-settlement growth (Figure 5).

### Post-larval Shell Ultrastructure and Elemental Composition: The Effects of pH

Oyster shell mineralization is mediated by cellular processes [91], and it seems that the structural and elemental compositions are strictly governed despite the environmental challenges. The present study exhibited juvenile oyster shell features that were well preserved in their overall thickness, morphological features and composition despite their formation under an extreme pH 7.4. The measured elemental contents of the calcareous shells produced by the juvenile *C. gigas* were statistically similar between pH treatments. When examined under an SEM, the crystallites formed under the extreme pH 7.4 showed no significant signs of impairment. Irrespective of pH treatments, these crystallites formed similar textural layers, i.e., outer granulated structures, which were possibly the remnant outermost layer as found in the larval shell and the inner foliated layer as seen in adult oysters [91], [92].

Although our current knowledge of the effects of pH on shell properties is very poor and depends solely on the few published data [73], [93], [94], [95], [96], [97], we know that the elemental compositions (i.e., Mg/Ca and Sr/Ca ratio) of shell minerals are sometimes affected by pH [98]. In contrast to the aforementioned hypothesis and similar to our results, the elemental incorporation into a coral reef was found to be unaffected by pH reduction [99]. However, the decreased pH (indicated as carbonate ion saturation) suppressed the incorporation of both Mg and Sr into foraminifera shells [100]. To maintain such a normal shell integrity and mineral composition, the juvenile oyster may endure a significant energy burden.

### Conclusions

Using a 40-day pH perturbation experiment, this study illustrated the potential resistance, tolerance and compensatory capacities of multiple life stages of the introduced Pacific oyster (*Crassostrea gigas*) to moderate and extremely decreased pH. Through multiple measurements of parameters including larval development, physiology and shell composition, an integrated view of larval response to pH exposure was achieved. The pre- and post-settlement growth parameters clearly remained unaffected by the today’s extreme decreased pH (pH 7.7) and the projected future average (pH 7.4). The weight-specific respiration and filtration rates of pediveligers decreased slightly at pH 7.4, although these measurements were statistically similar to the control pH (8.1) when normalized to the number of individuals. Such similar pre-settlement growth and larval feeding histories ensured normal metamorphosis without a significant delay. Furthermore, juvenile oyster shell ultrastructures and elemental compositions are the same irrespective of pH, suggesting that juvenile oysters will have normal protective armor under near-future decreased pH levels. Overall, our results suggest that the larval forms of the Pacific oyster population in the Yellow Sea may be pre-adapted to decreased pH compared with Japanese, American, Australian and European populations.
